# Hospital residents’ perception of mentorship as influencing clinical leadership: a qualitative study using semi-structured interviews

**DOI:** 10.12688/mep.19364.1

**Published:** 2023-02-03

**Authors:** Patricia McWalter, Abdullah AlKhenizan, Marium Ahmad

**Affiliations:** 1 Keele University, Keele, Newcastle, ST5 5BG, UK; 2 Department of Family Medicine and Polyclinics, King Faisal Specialist Hopital and Research Center, Riyadh, 11211, Saudi Arabia

**Keywords:** Mentorship, Clinical leadership, Hospital residents, Mentor, Supervisor, Leader, Role Model

## Abstract

**Background:** In this study, we explored how doctors in training perceive mentorship and leadership and whether they believed that mentoring influences the development of leadership skills. The study also addressed whether certain leadership styles lend themselves better to mentoring.

**Methods:** A qualitative research method was employed in this study and ethical approval was granted by the Research Ethics Committee (REC) at King Faisal Specialist Hospital and Research Centre (KFSH&RC), after which twelve hospital residents were recruited using purposive sampling. Semi-structured interviews were conducted by the authors and thematic data analysis was performed.

**Results:** Three themes emerged and were later refined, using Braun and Clarke’s 2006 thematic analysis method:

1. Purpose of mentorship, with sub themes: a. Expectations, b. Perception of mentorship as supervision, and c. The role of mentorship, including informal mentoring in leadership development

2. Role of mentorship in leadership development.

3. Perceptions of a leader, with sub-themes: a. The leader as a manager, b. The leader as a role model, and c. The merits of different leadership styles.

**Discussion:** Most of the residents (doctors in training) viewed mentorship in a positive way. However, when the mentor was perceived more as a supervisor, the usefulness of mentoring was less clear. However, they found that informal mentoring would contribute to leadership skills and would inspire them to become leaders themselves. They were likely to be influenced positively when they saw the leader as a role model, rather than a manager.

## Introduction

 There are many different interpretations of mentoring, with a blurring of the concepts of mentorship, supervision, assessment, coaching, and counselling. The authors infer that residents preferred the informal curriculum in acquiring clinical leadership skills, so there is a need for change in the current mentoring programme.

Healthcare services are better when doctors participate in leadership ( Harte & McGlade, 2018). However, there is a need for clinicians to be encouraged and supported in leading within the workplace ( McKimm & Swanwick, 2011). Mentorship, especially informal mentoring, is an important way of acquiring leadership skills ( Stamm & Buddeberg-Fischer, 2011). The word ‘mentor’ originates from Greek literature, meaning the trusted adviser to the son of Odysseus, as seen in Homer’s epic the ‘Odyssey’ ( Dugan & Fredell, 2000). The Saudi Arabian residency training programme puts emphasis on knowledge and skills competencies, rather than leadership development, but clinicians are now expected to participate in leadership roles and there is an increasing consensus that leadership skills should be developed in work-based activities. A recent study on mentoring medical students in Saudi Arabia found that clinical year students valued academic mentoring and had expectations that it would positively impact academic performance and career development ( Fallatah *et al.,* 2018). There is also limited research on the role of the informal curriculum and informal mentoring in the medical education literature ( Nicol & Gordon, 2018). The training programme for residents (doctors in training) in Saudi Arabia is conducted over four years and mentorship can either be formal, where mentoring is incorporated within an official programme or it can be more of an informal approach, which is unstructured and spontaneously organised. More recent conceptions of mentorship aim at breaking down traditional views of mentoring, and the trainee is encouraged to drive their own learning. Modern mentoring literature often focuses on getting the trainee to figure out what they need and recognises that the mentoring process should be individualised according to the mentee’s developmental needs ( Havard & Baker, 2018). Scholarly literature makes a point that mentors do not need to be more knowledgeable in the domain of mentorship but require other skills to do with facilitating learning about that domain ( McCrossan *et al.,* 2020). In study by Ong *et al.* a positive association was seen between mentoring and better postgraduate training including exam success and career progression, especially if the mentor was seen as a facilitator ( Ong *et al.,* 2018). A study from Malaysia highlighted the multifaceted nature of mentorship, for example the role of coaching in talent development, especially if the atmosphere is supportive and friendly ( Subramaniam *et al.,* 2015). Malaysia has a similar cultural environment to the Middle East, and the importance of having a supportive clinical learning environment within which mentoring takes place, is emphasised in this Malaysian study.

Interest has grown in the field of clinical leadership over the last 10 to 15 years, making this a timely study. Many countries are encouraging ‘a leadership culture among their clinical workforce’ and in some countries, physicians often work as health ministers, as politicians or in non-governmental organisations ( Coltart *et al.,* 2012, p. 1847). In the UK, the General Medical Council (GMC) has emphasised the importance of leadership for clinicians (General Medical Council, 2015). In Canada, physician training has emphasised the development of competencies in leadership ( Grady *et al.*, 2018). The Royal Australasian College of Physicians have incorporated leadership into the core competencies framework for physicians ( Udemans *et al.,* 2018). Leadership and fellowship programmes with a strong emphasis on mentorship have been set up in the USA and the UK (Darzi Fellowships), to encourage leadership development for residents. Short focused mentoring in the Darzi fellowship programme, found that informal mentoring by different mentors with high emotional intelligence was useful in facilitating the residents towards leading in the future ( Warren & Carnall, 2011). It is recognised that effective clinical leaders need support and nurturing within their health systems along with opportunities for mentorship, networking and experiential learning ( Swanwick & McKinn, 2011). Mentorship could guide junior doctors on their leadership journey and future clinical leaders can be prepared for leadership roles by positive role models ( Kiesewetter *et al.,* 2013). One of the key changes in clinical leadership styles over the years, has been the move towards a more dynamic and flexible leadership style, which has been shown to align better with complex leadership in healthcare ( Sonnenberg *et al.,* 2018). Globally, there is a move to encourage doctors in specialty training to gain “medical expert competence”, in addition to leadership, communication and organisational skills ( Malling *et al.,* 2020, p.1).

This research presents a novel approach as the first study from Saudi Arabia examining the role of mentorship in the development of leadership skills for residents. This study explored how doctors in training perceive mentorship and leadership and whether they believed that mentoring influences the development of leadership skills. The purpose of the study was to establish if there was a link between mentorship and leadership, specifically if mentorship contributed to the development of leadership skills and if certain leadership styles lend themselves better to mentoring?

Some limitations of this study include the fact that it was carried out at a single centre in the Middle East, which may make it less generalisable to a global setting. King Faisal Specialist Hospital and Research Centre (KFSH&RC) is a large centre so the study may not be applicable to smaller centres or practices. However, the institute employs staff from over sixty different countries. The residency programmes only accept doctors who graduate from Saudi universities so only Saudi residents were interviewed, . Their experiences therefore came from the Saudi educational and healthcare systems. However, some of their mentors and supervisors had training from the UK, the USA, and Canada. Twelve residents were interviewed but they came from various hospital departments, adding diversity to the study. The residents were in the final two years of their residency programme. First and second year residents were excluded as we felt they may not have had adequate exposure to mentorship and leadership. As quantitative data, such as demographics and the percentage of residents who progressed into leadership roles, was not collected, we do not have further information that could have enriched the study, regarding the influence of mentorship on leadership. In order to have learnt more about global healthcare leadership development, perhaps interviewing more senior doctors with experience from Saudi Arabia and other countries would have been enlightening.

Most of the residents viewed mentorship in a positive way, particularly if informal in nature, rather than supervisory. The informal curriculum was more likely to lead to the development of leadership skills and residents were inspired, when they saw their leaders as role models, rather than managers. 

This study will look at the expectations and perceptions of residents in relation to mentorship and leadership and whether they believe mentoring influences the development of leadership skills. Finally, we will explore the resident’s perceptions of a leader and whether certain leadership styles lend themselves to being a better mentor.

## Methods

### Study setting

In December 2020, the researchers carried out twelve individual, face-to-face, semi-structured interviews with hospital residents at King Faisal Specialist Hospital and Research Centre (KFSH&RC), Riyadh in Saudi Arabia, which is one of the most prestigious research hospitals in the Middle East. The researchers were all Consultant Family Physicians (MD) at KFSH&RC, comprising two female doctors and one male doctor. They all had experience in research and teaching, having completed Master’sprogrammes in Medical Education. The interviews were conducted by the two female researchers. The sample interviewed came from a diverse range of specialties, including Family Medicine, Paediatrics, Internal Medicine, Surgery and Obstetrics and Gynaecology and they were in the third or fourth year of their residency training programmes (R3 and R4).

### Sampling and recruitment

A total of twelve participants were recruited using purposeful sampling, where the most productive sample is selected, to answer the research question. The programme directors of the various specialties were contacted to make recommendations on which residents in the final two years of their training, should participate in the study, and some of those residents suggested other residents, the so-called “snowball sample” ( Marshall, 1996, p.523). The twelve residents all agreed to participate and were interviewed face to face, using a semi-structured interview approach. There was six male and six female participants. Four of the participants were known to the interviewers as they were working in the same department (Family Medicine). The remaining eight participants were not known to the interviewers, other than through email and telephone communication prior to the interview, to get initial consent and arrange a time and venue for the interview. The participants were informed about the researcher’s clinical and academic positions. All the participants, including the four residents from the same department as the researchers, were unaware of the researcher’s specific research interests or views on the research topics.

### Interview schedule

The questionnaire was divided into four parts; the first part addressed perceptions of mentorship, moving onto questions on leadership, and later discussion around the influence of mentoring on leadership, finishing up with the leadership styles most suited to mentoring. Audio recordings were used to collect data from the interviews and the questionnaire guided the semi-structured style used for all the interviews. The interviews were conducted over a six week period and the analysis took a further six weeks.

### Design

A qualitative approach, epistemologically grounded in social constructionism was employed in this study. This design is suited to the exploration of the residents’ perceptions of mentorship and leadership in this study and how the two concepts are linked. The authors became familiar with the transcribed data by repeated and active reading of the data, and checking the transcripts against the original audio recordings, thereby increasing accuracy and transparency ( Varpio & Kieger, 2020). Initial codes were generated, and an inductive coding framework developed, allowing for an audit trail, which increased the trustworthiness of the interpretation ( Braun & Clarke, 2006). The next steps involved searching for broad themes and later reviewing the themes, moving onto defining and naming the themes and sub-themes. Thematic analysis following Braun and Clarke’s six-step approach, as shown in Table 1, offers a clear guide for doing thematic analysis ( Braun & Clarke, 2006). The themes were mainly descriptive, in that patterns in the data, relevant to the research question, were highlighted ( Maguire & Delahunt, 2017). After the interview was conducted and transcribed, participants were provided with access to the transcribed interview and were informed they could amend any aspect of this, ensuring the transparency and trustworthiness of the data analysis. The interview recordings and transcripts were saved on one file in my OneDrive system, which was encrypted to ensure all data was kept in a secure location.

**Table 1.** Braun & Clarke’s six-phase framework for doing a thematic analysis ( Braun & Clarke, 2006)

Step 1: Become familiar with the data	Step 4: Review themes
Step 2: Generate initial codes	Step 5: Define themes
Step 3: Search for themes	Step 6: Write-up.

### Ethical considerations

This research was granted ethical approval in November 2020 by REC, the review board for KFSH&RC, which was the granting institute for the principal investigator (Protocol number: C380/284/42).

### Consent

Prior to conducting interviews, the researcher received informed consent from the participants. Participants were provided with an information sheet which detailed the aims and objectives of the research, along with information about the interview (general information being sought and approximate length of the interviews). Participants were informed that they had the right to withdraw at any stage of the research process. They were also provided with a consent form, which they were asked to complete and return to the researcher. The interviews did not commence before the consent form was returned. All participants contacted at the start of the research study signed the consent form and agreed to be interviewed for this research study. Prior to the commencement of the interviews, participants were asked to orally verify that they were satisfied to proceed with the interview and consented to the recording of the interview. They were assured they could terminate the interview at any stage. 

## Results

The residents came from different specialties including family medicine, surgery, internal medicine, paediatrics and obstetrics and gynaecology. The three main themes which we had already expected were refined into: 1. Purpose of mentorship, including expectations from mentoring, perception of mentorship as supervision, and value of informal mentorship; 2. Role of mentorship in leadership development, including the linking of mentorship and leadership (or not); and 3. Perceptions of leadership, including leader as a manager, leader as a role model, and merits of different leadership styles.

### 1) Purpose of mentorship

The mentoring process was generally regarded as supportive, and most residents had a positive attitude towards mentorship. Some residents met regularly with their mentor, usually every one to three months. Overall, the mentor was seen as a kind person, who embodied professionalism, integrity, generosity and wisdom. The trainees valued the personalised interaction with their mentor and good communication formed the basis of the mentoring process. The residents saw the mentoring process as a way to improve their practice and critical reflection and one resident felt that it was one of the most important factors in progression through the residency programme. Some felt more confident to deal with difficult situations in their practice after the mentorship process and believed that it shaped them, as well as helping with professional development. Others saw mentoring as a way of guiding them towards a path that they were passionate about. One resident who loved mentorship highlighted that their mentor had a background in Cognitive Behavioural Therapy (CBT), indicating that this resident saw mentorship as a type of counselling, helping her through difficult times:

‘Having someone to talk to about the difficulties you’re going through in the programme’ (R10).

Although the concept of mentoring was viewed as important, the mentorship process in its current form was perceived to not always be useful. The focus was on acquiring competencies like clinical knowledge and skills during the residency programme. Lack of time was seen as a barrier to a proper mentoring process and involved much paperwork.

'I don’t think it was like proper mentoring …I don’t like the papers. It feels more like a chore’ (R11).

***a) Expectations from mentorship***

The residents had a wide range of expectations from the mentoring process. Residents wanted their mentor to be accessible, cooperative, flexible, non-judgmental, knowledgeable, and experienced; showing them the right path. Others believed that academic knowledge was not so important, but instead the mentor should have knowledge about the mentee, their specialty, the institute, and the programme. The mentor, in one instance, was approached by the trainee to support them with applications and research. Some residents had expectations of a more meaningful mentoring experience. They expected the mentor to help them handle situations better, enabling them to focus on the big picture.

‘I don’t need the person’s knowledge or my mentor’s knowledge…I just need them to listen and to understand… I can go to any consultant and ask them for their knowledge about the specific subject’ (R10).

25% of the residents preferred to have a mentor of their own choosingwho would help them grow. There was a widely held opinion that every resident needed a mentor.

***b) Perception of mentorship as supervision***

The concept of supervision was prominent in the residents’ interpretation of mentorship, with emphasis on assessment, feedback, evaluation, and reflection. One of the residents valued being in the clinic with his mentor at least once per week, in order that knowledge and skills could be assessed, emphasising the view that mentorship was linked to assessment. It was noted in some responses that constructive feedback helped the residents improve themselves, especially in areas like communication, time management skills, reflective practice and professional development.

‘I like that there is an open option to talk to someone, who is more knowledgeable or more experienced’ (R6).

Some residents did not view the mentoring sessions as proper mentoring and others found that the sessions did not allow for critical reflection as so much paperwork had to be signed. There was also a view amongst residents sampled that clinical reflection was part of their daily work and writing this down as part of mentorship was not required, as residents were already doing this.

***c) Role of Informal mentorship***

Informal mentorship was highly valued by the residents (a total of 10 out of the 12 sampled) and it was considered important to have a mentor who was approachable and available. There was a belief amongst residents that a mentor is just ‘born with it’ and has an innate ability to mentor. Two residents did not meet the mentor assigned to them but utilised other sources of support. This informal mentor was often a senior colleague and was admired because of their knowledge and how they treated their patients. Another resident mentioned a preference to keep mentoring more informal and friendly and did not want to formalise the process. Some of the key people who had positively impacted the residents were informal mentors, for example family members or teachers in school and university, as well as doctors and professors who had influenced their career choices. One resident identified the University Medical Dean as the person who had encouraged them to pursue a career in surgery. Some came from a family of physicians and found their informal mentoring there:

‘Another form of informal mentorship happened when I was a child…the support of my father’ (R8).

### 2) Role of mentorship in leadership development

Most residents felt that mentorship was important in leadership development, and one resident found that mentoring had given them the confidence to pursue a leadership role.

‘When you are picked by your consultant or the head as a mentor or as a leader, maybe this gives you more confidence in yourself’ (R12).

With mentorship, another trainee felt empowered to implement change and be productive in the future. When constructive feedback was part of the mentoring process, some residents were guided into teaching and leadership roles.

However, the majority felt that they did not get adequate exposure to leadership opportunities during their residency. Leadership was not part of the curriculum in the Saudi Residency training programme at the time of conducting this study and leadership was rarely included in the mentoring discussions held as part of the official training programme.

‘I don’t think that we are getting enough chances in terms of leadership’ (R1).

***a) Linking mentorship and leadership (or not)***

According to most of the residents interviewed, a good leader was not necessarily a good mentor. However, these qualities might reasonably coincide in the same person and if a good leader was also a good mentor, this would likely have a positive impact on the trainee. Half of the residents felt that mentorship had helped them in their leadership aspirations, and some residents mentioned that people at their medical school had guided them into leadership roles, supporting them with communication skills. Discussion on leadership sometimes occurred during the mentoring session and one resident felt that leadership training should be incorporated into the residency programme, including lectures about leadership. Most thought that mentorship and leadership should be interlinked, and most would feel confident going into leadership roles, especially after informal mentoring,

For those who did not feel mentoring was linked to leadership, they saw the mentoring process as an act, rather than an ongoing relationship and in this form; it did not encourage residents into leadership. Another commented that linking the two concepts was difficult as a mentor should be empathetic but sometimes a leader needed to be firm.

‘I did have leadership roles in my med school and in my internship here. It doesn’t seem right yet. I think in the future maybe…… I don’t think the mentorship programme at our department will definitely change anything about my leadership course’. (R7)

### 3) Residents’ perceptions of leadership

A total of 83% of residents viewed clinical leadership as the ability to perform clinical duties and also to lead, with a perception of the leader as a facilitator. The model clinical leader was perceived by the residents as a good communicator, patient, friendly, resourceful, and polite, with knowledge and experience in leadership. It was acknowledged that leadership was easier if the leader had the skills and knowledge of the situation and if they were supported by co-workers, and management above the leader. The ideal clinical leader would ask for feedback, in order to improve themselves and through their knowledge of the strengths and weaknesses of their team, they could make improvements in the team. Some believed that formal training in leadership helped in the development of skills, while others viewed leadership skills as innate:

‘I don’t think anyone should take any leadership courses…. He should be with his team. To support the team rather than point out certain jobs to do’ (R7).

‘You have a team who are invested…all the team working together…people are a major factor’ (R12).

***a) Leader as a manager***

Some of the residents saw a leader as one who was in charge of the department, serving the team as a manager, with the residents following his or her lead. The leader, according to these participants, took care of the team, made them feel comfortable and endeavoured to do the right thing. The leader had a clear vision, to ensure the success of the group:

‘Leadership is to be able to put a plan, to be able to manage, to be able to have your own vision or your own goals as you will be able to achieve this goal’ (R7).

The leader as a manager also faced many challenges, including time pressure, stress, trying to make everyone happy, conflict in the team, and lack of support.

***b) Leader as a role model***

Some viewed the leader as a role model, who led by example. The leader in this context showed the residents the right path and they were admired because of the way they treated their patients and staff. They taught the residents good values and how to be critical thinkers. The leader was humble and honest, with good morals and was someone to follow. The residents were inspired to follow the senior consultant, and senior residents if they had a passion to help and a desire to pass their talents onto future generations. The doctor was seen as the cornerstone of the medical team and the residents aspired to be like the physician leader.

‘I like to work with the team, not give orders…I like to have a personal relationship with my team and to make friends before I even make the team. That’s one of the arts in achieving the goals that I have’ (R5).

‘He would tell me if I were in your shoes, I would do 1,2,3,4,5, so next time I would definitely take that into perspective……. Should be an example by his own character’ (R7).

***c) Merits of different leadership styles and influence on mentorship***

The clinical leadership styles discussed in this study were hierarchical, distributive and situational. Those who preferred the hierarchical style saw the leader as building improvement, with the team following. They share the leader’s vision, and conflict is avoided. Some residents liked the concept of following the leader when it came to mentorship:

‘It’s important to me that I see people do such things for me to follow’ (R12).

For others, the distributive style was preferred, as there were more personal interactions, leading to better team dynamics and better outcomes. The trainee felt empowered by this dynamic and perceived this as an effective means to improve their skills:

‘Come up with a decision together…team approach’ (R10).

Some also felt a distributive style lend itself best to mentorship, and believed that the good leader was also a good mentor to the team, where they could:

‘Empower the trainee and improve their skills’ (R5).

A few residents felt that the leadership style adopted by a mentor depended on the situation and the people involved.

## Discussion

The results of our study highlight a lack of consensus as to what exactly mentorship constitutes. Some residents viewed mentorship as supervision and leadership as management. This could be explained by the strong emphasis placed on acquiring knowledge and skill competencies during medical school and residency training. During the residency training programme in Saudi Arabia, the Saudi Commission for Health Specialties ( SCFHS, 2022), which is equivalent to the General Medical Council (GMC) in the UK, requires residents to fulfil competencies, especially in clinical knowledge and skills. In this study, the mentor, as a supervisor, provides assessment, feedback, evaluation and encourages reflection, as well as helping the residents with time management, communication skills and professional development. The mentor was seen by many participants as someone who should possess a higher knowledge, wisdom and experience, often suggesting a hierarchy. Some of the residents in this study expressed an opinion that mentorship in its current form would not inspire them to pursue leadership opportunities.

 Some residents in our study recognised the facilitatory role of their mentor. A few residents in our study had views on mentorship in keeping with modern scholarly literature. Although most of the trainees felt more positive about mentorship after the mentoring experience, some felt that the current programme did not allow for full professional development and is not based on modern conceptions of mentoring. Establishing mentoring relationships in the workplace has been found to support junior doctors with career choices, as well improving staff morale and leading to better patient care ( Powell *et al.*, 2018). In our study, the residents also valued informal mentorship in their everyday clinical work, as well as support with their career plans and professional pursuits including leadership and research.

In our study, the residents favoured a friendly environment during their mentoring session. In our study, trainees valued mentors who were kind, professional and friendly and gave advice if the residents were going through difficult times. According to Launer (2014, p. 111) Junior doctors expressed that ‘supervision, coaching, or mentorship may have been the most important part of their learning’. One resident viewed mentoring as an opportunity to overcome difficulties, rather than, for example, to support potential that needs to be developed. He saw his mentor as one who supported him with problems and helped him to avoid stress and burn-out. This mentoring supervisory style is more in keeping with a traditional apprenticeship approach, where the supervisor is the dominant one. On the other hand, an issue identified in this study was the challenge of setting up dedicated formal mentoring sessions, with two residents reporting never meeting their assigned mentor. One study highlighted that the availability of adequately trained faculty may be a challenge for training programmes ( Sonnenberg *et al.,* 2018). In our study, it was highlighted that the demands of clinical work and excess paperwork were getting in the way of having a proper mentoring experience, despite acknowledging the need for every resident to have a mentor.

Regarding leadership learning, one study discussed General Practitioner (GP) trainees’ views on tutorials, partnership meetings, small group teaching, courses and multi-source feedback, as ways to learn about leadership. They tended to view leadership courses negatively, preferring informal leadership. The informal curriculum was considered to be valuable in developing leadership during residency and the informal mentor was viewed as having innate mentoring skills, with the resident more likely to see their mentor as someone to follow or as a role model ( Nicol & Gordon, 2018). This was similar to our study, in that the residents learned a lot from their supervisors and mentors, through role modelling and coaching. There is a need to prioritise leadership during medical school training and residency programmes. Over half of the residents interviewed in our study felt that leadership and mentorship should be linked, and informal mentoring had influenced some of the trainees to pursue leadership roles. A systematic review on leadership training in graduate medical education, found that ‘mentoring and coaching are valuable components of leadership curricula’ ( Sadowski *et al.*, 2018, p. 145) but there is no clear consensus on the best way to teach leadership skills. However, small group learning, mentoring and coaching were the most common modalities in the high-quality research. 

 In our study, some residents viewed the leader as a manager, where the team followed an effective leader who incorporated the idea that a team should be easy to lead, rather than the leaders having a role in developing such a team. Leadership, in this context, is about getting people to enact your plan and vision, and junior doctors have few opportunities to engage in leadership positions during their training. Regarding leadership styles, modern research alludes to the need for an adaptive leadership style depending on the situation. Gilbert’s situational theory emphasised that leaders have to adapt their style, depending on the situation and being successful in one field doesn’t necessarily translate to another area ( Gilbert, 2005). In our study, some residents felt that the leadership style adopted, depends on the situation and the individual leader. Saxena *et al.* (2017, p.2) believe‘that the most effective leaders use a wider range of leadership styles and choose the most appropriate style for a given setting’. In this study, some of the residents viewed the leadership style at the institute as more bureaucratic and hierarchical, and although a few supported this style, the majority expressed a desire for distributive or situational approaches. A distributive style focuses on achieving a ‘shared vision’ ( Martin *et al.,* 2015, p.16) and formed the basis of the NHS Leadership Academy, where there is a strong focus on teamwork and a pooled talent, which is a better approach to managing complex healthcare organisations ( Ellis & Abbott, 2014). In this study, the residents liked the idea of more personal interactions as well as coming up with decisions together as a team.

Regarding leadership training during residency, some residents in the study felt that leaders should empower the trainee and try to improve their skills in leading. Other residents mentioned that discussion about leadership occurred during informal mentoring sessions, and one resident felt that leadership training should be part of the mentoring process. One trainee suggested that lectures on leadership should be added to the residency curriculum, but this method is in keeping with a traditional way of learning. The updated Family Medicine curriculum in 2020 by the Saudi Board for Family Medicine in Saudi Arabia has now included management and leadership in the competency framework ( Al-Khaldi *et al.,* 2017), indicating progress towards focussing on leadership skills. There is a call by Medical Faculties to have more emphasis placed on experiential learning, rather than theory alone, as well as having communication and teamwork skills included in the residency leadership competency framework ( Maddalena, 2016). A total of four out of twelve residents in this study enjoyed shadowing their senior colleagues and viewed some of them as role models, again emphasising the importance of the informal curriculum. The role model leader was admired because of their attitude, their service and generosity of time to the students.

## Conclusions

The Mentorship programme in Saudi Arabia puts emphasis on knowledge and skills competencies, rather than leadership development, which is very similar to other countries like Canada, the USA and the UK. In this study, there were many different interpretations of mentoring, with a blurring of the concepts of mentorship, supervision, assessment, coaching, and counselling, thus making it difficult to set up a formal mentorship programme. The residents did not perceive the mentorship programme in its current form as influencing clinical leadership in the future. In conclusion, most residents preferred the informal curriculum in acquiring clinical leadership skills, indicating a need for change in the current mentoring programme. There is often a tension as to what clinical leadership should stand for. In this study, leadership was often perceived as getting people to follow the leader’s plan and vision; the residents viewed their leaders more as managers. This lack of clarity on management and leadership roles is not unique to this study. Some residents saw the importance of a distributive leadership style in relation to being a better mentor, rather than using a hierarchical style. Leadership and management skills have recently been included in the competency framework of the Saudi Resident training programme. Similar to the NHS Leadership Academy in the UK, the Health Leadership Academy (HLA) in Saudi Arabia is encouraging the development of leadership in health systems, with the aim of helping trainees gain experience in the healthcare system ( SCFHS, 2017). There is a need for more research on mentorship in developing clinical leadership for doctors of all grades, but especially for junior doctors, where limited studies have been done. This research will help educators better understand how mentoring can help learners become leaders in the future and which leadership style lends itself best to mentorship.

• **Practice points**

•   The residency training programme puts emphasis on knowledge and skills competencies, rather than leadership development. But clinicians are now expected to participate in leadership roles and there is an increasing consensus that leadership skills should be developed in work-based activities.

•   From the literature, mentorship, especially informal mentoring, is an important way of acquiring leadership skills

•   Scholarly literature indicates that the most effective leaders use a wider range of leadership styles and choose the most appropriate style for a given setting.

## Ethics

This research was granted ethical approval in November 2020 by REC, the review board for KFSH&RC, which was the granting institute for the principal investigator (Protocol number: C380/284/42).

## Consent

Written informed consent for publication of the participants data was obtained from the participants.

## Data Availability

Repository name: Hospital Residents perception of Mentorship as influencing Clinical Leadership. https://doi.org/10.17605/OSF.IO/VUH2W ( McWalter, 2022) The project contains the following underlying data: Raw data - containsde-identified qualitative participant responses Repository name: Hospital Residents perception of Mentorship as influencing Clinical Leadership. https://doi.org/10.17605/OSF.IO/VUH2W ( McWalter, 2022) This project contains the following extended data: Data Collection Sheet pdf.pdf (questionnaire - contains blank template interview script) Data are available under the terms of the Creative Commons Zero “No rights reserved” data waiver (CC0 1.0 Universa).
