# Supplementary material for: An Evaluation of Mentorship for Hospital Residents in Saudi Arabia: A Qualitative study using Semi-structured Interviews
Source: MedEdPublish (2016). 2023 Oct 31;13:8. Originally published 2023 Feb 3. [Version 2] doi: 10.12688/mep.19364.2 (PMC10877095; doi:10.12688/mep.19364.2)
Supplement: Peer review history

# Reviewer report: Reviewer response for version 2

**DOI:** 10.21956/mep.21193.r35902

**Published:** 2024-02-19

**Authors:** Dr.Hamid Saeed

**Affiliations:** 1 British University in Dubai, Dubai, United Arab Emirates

The authors have addressed an important topic of mentorship and leadership in the healthcare specifically with reference to the Saudi context.

 The issue of disconnect between curriculum and professional requirements of having these skills by the medical professionals is highlighted to some extent.

 Background:

 The background of the study needs to be more convincing. The authors must highlight what issues prompted them to carry out this study. The lack of mentoring and leadership skills and their negative impact on the current practices should be mentioned with some evidence from the existing literature. Proofreading and better transition between the sentences is required for this section.

 Literature Review:

 This section needs improvement in terms of the logical flow of the information. The first paragraph is mixed with definitions and findings from the literature and is very fragmented. The mentorship definition is repeated twice in the section. The findings of the study should not be discussed in the literature review section. Proofreading and better transition between the sentences is required for this section.

 Methods:

 The interview schedule is repeated in the "Design" paragraph.

Have any limitations of the research been acknowledged?

Yes

Is the work clearly and accurately presented and does it cite the current literature?

Partly

If applicable, is the statistical analysis and its interpretation appropriate?

Not applicable

Are all the source data underlying the results available to ensure full reproducibility?

Yes

Is the study design appropriate and is the work technically sound?

Partly

Are the conclusions drawn adequately supported by the results?

Yes

Are sufficient details of methods and analysis provided to allow replication by others?

Partly

Reviewer Expertise:

Leadership and innovation in healthcare

I confirm that I have read this submission and believe that I have an appropriate level of expertise to confirm that it is of an acceptable scientific standard, however I have significant reservations, as outlined above.

# Reviewer report: Reviewer response for version 2

**DOI:** 10.21956/mep.21193.r35589

**Published:** 2024-01-24

**Authors:** Stephanie Ewuzie

**Affiliations:** 1 Warwick Business School, Warwick, UK

The results of the study are interesting. mentorship has not been explored much in a non-western context, so I think this is a necessary study. you point out the limitations of the study - one research site. this is good.

 However, the structure of the article needs more work.

 Background/introduction:

 This section does little to ground the study. you may want to discuss the purpose of this study, what puzzle you would like to address, or what gap in literature you would like to fill. this section also lacks definitions of important concepts.

 Literature review:

 You may want to revisit this section. although some past studies are mentioned, you also reported some of your findings here. *“We had a similar finding, which may also be explained by the relatively recent introduction of mentorship into the Saudi curriculum.”*

 In another part you stated: " *Most of the residents viewed mentorship in a positive way, particularly if informal in nature, rather than supervisory. The informal curriculum was more likely to lead to the development of leadership skills and residents were inspired, when they saw their leaders as role models, rather than managers. The authors infer that resident preferred the informal curriculum in acquiring clinical leadership skills, so this needs to be addressed in the newly established mentorship programme*."

 In this section, you also stated: There is limited research on the role of the informal curriculum and informal mentoring in the medical education literature ( Nicol & Gordon, 2018). (Is your study trying to cover this gap?)

 The authors need to read through the work to separate their findings from existing scholarship in the literature review.

 This may help with relevant references: [1]

 Lastly, proofreading is necessary as this was repeated twice in the document *"The word ‘mentor’ originates from Greek literature, meaning the trusted adviser to the son of Odysseus, as seen in Homer’s epic the ‘Odyssey’ ( Dugan & Fredell, 2000)*.

 Methods

 The authors did well to discuss the meaning of the deductive approach. 

 the authors used the word "questionnaire" did you mean an interview question guide?

 Findings:

 Interesting findings however, more showing of data would have helped to reveal the tensions that participants encountered in the mentoring program. for instance, " *Some residents did not view the mentoring sessions as proper mentoring and others found that the sessions did not allow for critical reflection as so much paperwork had to be signed."*

 This is an interesting tension/puzzle to explore. what were the results of the paperwork? who did it go to?

 Discussion

 it would be more interesting to begin this section with a summary of the research. 

 Lastly, you may want to think about how this adds to the current literature on mentoring. In other words, clearly state your contribution to scholarship.

Have any limitations of the research been acknowledged?

Yes

Is the work clearly and accurately presented and does it cite the current literature?

No

If applicable, is the statistical analysis and its interpretation appropriate?

Not applicable

Are all the source data underlying the results available to ensure full reproducibility?

No source data required

Is the study design appropriate and is the work technically sound?

Partly

Are the conclusions drawn adequately supported by the results?

Partly

Are sufficient details of methods and analysis provided to allow replication by others?

No

Reviewer Expertise:

Professions, emotions, professional socialisation in health care, health care systems, Global South research.

I confirm that I have read this submission and believe that I have an appropriate level of expertise to state that I do not consider it to be of an acceptable scientific standard, for reasons outlined above.

## References

[1] : Seeing You in Me and Me in You: Personal Identification in the Phases of Mentoring Relationships. *Academy of Management Review* .2016;41(3) : 10.5465/amr.2013.0203 435-455 10.5465/amr.2013.0203

# Reviewer report: Reviewer response for version 1

**DOI:** 10.21956/mep.20750.r33898

**Published:** 2023-08-03

**Authors:** Iain Snelling

**Affiliations:** 1 Health Services Management Centre, University of Birmingham, Birmingham, England, UK

This is an interesting paper, but it covers a lot of ground thinly.

 The study explores four issues as described in the introduction.

- expectations and perceptions of residents in relation to mentorship and leadership
- the outcome of mentoring, specifically related to the development of leadership skills.
- resident’s perceptions of a leader
- the relationship of leadership styles to effective mentorship.

These are substantial questions in their own right, and discussing them together seems to conflate a number of issues and add little depth to what is already in the literature. The introduction explores a number of areas of relevant literature, including trainees’ learning about leadership; mentoring, informal and formal, in training programmes; clinical leadership which seems to relate mainly to leadership in a formal sense; and at the end of the second paragraph the 'cultural environment' is considered. These areas are considered briefly, with the result that key issues (some of which are reflected in the data) are not fully identified.

 The research seems to be an evaluation of a mentoring programme. The context of the experiences of mentoring that are being explored is not explained. For example: is this a new scheme, or an established one. Who are the mentors, and how were mentors and mentees matched? Do trainees also have Educational Supervisors with a formal role? What are the requirements of the mentor relationship? Some ‘paperwork’ is required (suggesting a formal scheme), but informal mentoring is also highlighted. The current focus of the mentoring programme is ‘on acquiring competencies like clinical knowledge and skills’. The consideration on leadership learning in mentoring is therefore not clear. Is there an expectation that mentoring in the programme will include development of leadership which has recently been added to the Resident training programme?

 Are mentors leaders, in a formal sense? If there was a focus on leadership in the scheme, that might make sense, but it seems that there is not. The link between leadership styles and mentoring is not clear in the research question. Do the mentees experience the mentors leadership outside of the mentoring relationship (which would give theme clear view of leadership style).

 There is some useful discussion of mentoring in the literature review, but it isn’t clearly defined, which may be important as other supporting roles are also identified in the article such as supervisor, and leader. One of the conclusions of the paper is that there is a blurring of these concepts (and of coaching, assessment, and counselling) in practice. Is there greater conceptual clarity in the literature? The idea of the ‘informal curriculum’ might have been explained in a little more depth. Would that include ‘informal mentoring?’ Does it relate to ideas of the ‘hidden curriculum’?

 Clinical leadership (or it might be relevant to think specifically of medical leadership) is considered in the introduction from the point of view of position. Leadership in a distributed sense as an element of medical practice is not highlighted, although was suggested in the data. The GMC has recently (2017) published the Generic Professional Capabilities framework which has now been implemented in all postgraduate curricula. The GMC document referred to in the paper (2015) is not in the reference list, but the GMC has emphasised for many years that leadership is a requirement for all doctors.

 The design of the research is deductive, and so a more comprehensive exploration of relevant concepts at the start would be helpful, with a consideration of how clarity in key areas contributed to the research, for example in how the interviews were conducted or data was analysed.

 The lack of a clear conceptual focus in the questions, and the wide breadth of issues means that the results are of limited benefit. If this was reframed as an evaluation of the current scheme, then they would make more sense, and could be seen more developmentally with perhaps some implications for improving the role of mentorship. At times in the data it is not clear whether interviewees are talking of their experiences of the current mentoring programme, or more generally – for example ‘a good leader was not necessarily a good mentor’. In the first part of the results, the expectations from mentorship, seem to be more expectations of a mentor. Similarly, in the section on Residents’ perceptions of leadership, this seems to be about leaders rather than leadership. This adds to the lack of clarity on the questions. Some quantitative data from the interviews is included, through classifying interview data. A survey might have been useful as part of the design – the design should be justified more clearly.

 The second sentence of the discussion sums up some of the lack of clarity: “Some residents viewed mentorship as supervision and leadership as management.” There are two distinct issues here. Later it says that “leadership and mentorship should be linked”. Does this mean that mentoring should be focussed on leadership development, rather than clinical skills development? Or that mentors should be leaders, presumably in a formal role? Should leadership development mentoring be separate from mentoring for clinical skills development, which I think is the main current focus? These are examples of the lack of clarity in the results.

 The specific Middle East context was highlighted in the introduction, but wasn’t subsequently managed. Evidence from different contexts is useful, and so this aspect might be developed. The lack of detailed contextual information was commented on above.

 The first sentences of the conclusion imply a evaluation aim with a focus on the current programme. I think it would be useful to reframe the paper as an evaluation of the current mentoring programme, if indeed this was the aim.

 I think that the introduction should be more comprehensive in outlining the current scheme and the range of issues being considered. The lack of clarity here is followed through into the results. A clearer focus might be possible to add depth, perhaps at the expense of breadth.

 To comment on the specific review questions:

- *Is the work clearly and accurately presented and does it cite the current literature?*
  Assessed as partly because the literature review does not fully explore the relevant concepts in the literature.
- *Is the study design appropriate and is the work technically sound?*
  Assessed as partly. A qualitative design is appropriate, but a survey might have been considered. Some qualitative data is expressed quantitatively. The deductive nature of the design might be explained more clearly.
- *Are sufficient details of methods and analysis provided to allow replication by others?*
  Assessed as No, because details of the educational context in Saudi Arabia are not provided. A copy of the interview schedule might be useful.
- *Have any limitations of the research been acknowledged?*
  Yes, but this might have been more critical – for example in not exploring the views of mentors, and in the sample size which seems quite small.

Have any limitations of the research been acknowledged?

Partly

Is the work clearly and accurately presented and does it cite the current literature?

Partly

If applicable, is the statistical analysis and its interpretation appropriate?

Not applicable

Are all the source data underlying the results available to ensure full reproducibility?

Yes

Is the study design appropriate and is the work technically sound?

Partly

Are the conclusions drawn adequately supported by the results?

Yes

Are sufficient details of methods and analysis provided to allow replication by others?

No

Reviewer Expertise:

Medical leadership, and medical leadership development.

I confirm that I have read this submission and believe that I have an appropriate level of expertise to confirm that it is of an acceptable scientific standard, however I have significant reservations, as outlined above.

# Reviewer report: Reviewer response for version 1

**DOI:** 10.21956/mep.20750.r33896

**Published:** 2023-07-24

**Authors:** Kathy Chappell

**Affiliations:** 1 American Nurses Credentialing Center, Silver Spring, Maryland, USA

Overall, a nice qualitative study exploring the concept of mentorship from the perspective of Saudi physicians in training.

 Few comments: 

- The author switches from past tense, to present, to future tense. As this is a completed study, would recommend all be in past tense.
- Nice job on recent literature citations.
- Should the fact that both interviewers were female be considered as a limitation in this context?

-

Have any limitations of the research been acknowledged?

Yes

Is the work clearly and accurately presented and does it cite the current literature?

Yes

If applicable, is the statistical analysis and its interpretation appropriate?

Yes

Are all the source data underlying the results available to ensure full reproducibility?

Yes

Is the study design appropriate and is the work technically sound?

Yes

Are the conclusions drawn adequately supported by the results?

Yes

Are sufficient details of methods and analysis provided to allow replication by others?

Yes

Reviewer Expertise:

Interprofessional education; accreditation of IPE and CNE; accreditation of residency and fellowship programs

I confirm that I have read this submission and believe that I have an appropriate level of expertise to confirm that it is of an acceptable scientific standard.

# Response

**Published:** 2023-08-07

**Authors:** patricia mcwalter

**Affiliations:** King Faisal Specialist Hopital and Research Center, Saudi Arabia

Thank you for the feedback. Much appreciated
